# Enhancing Antioxidant Benefits of Kombucha Through Optimized Glucuronic Acid by Selected Symbiotic Fermentation Culture

**DOI:** 10.3390/antiox13111323

**Published:** 2024-10-30

**Authors:** Yu-Chieh Chou, Hui-Wen Lin, Chung-Yi Wang, Chen-Che Hsieh, Shella Permatasari Santoso, Shin-Ping Lin, Kuan-Chen Cheng

**Affiliations:** 1 Ph.D. Program in Drug Discovery and Development Industry, College of Pharmacy, Taipei Medical University, 250 Wu-Hsing Street, Taipei 11031, Taiwan; 2 Institute of Medicine, Chung Shan Medical University, Taichung 40201, Taiwan; 3 Institute of Food Science and Technology, National Taiwan University, 1 Roosevelt Road, Sec. 4, Taipei 10617, Taiwan; 4 Department of Seafood Science, College of Hydrosphere, National Kaohsiung University of Science and Technology, Kaohsiung 81157, Taiwan; 5 Department of Chemical Engineering, Widya Mandala Surabaya Catholic University, Kalijudan 37, Surabaya 60114, East Java, Indonesia; 6 Collaborative Research Center for Zero Waste and Sustainability, Jl. Kalijudan 37, Surabaya 60114, East Java, Indonesia; 7 School of Food Safety, Taipei Medical University, 250 Wu-Hsing Street, Taipei 11031, Taiwan; 8 TMU Research Center of Biomedical Device, Taipei Medical University, 250 Wu-Hsing Street, Taipei 11031, Taiwan; 9 TMU Research Center for Digestive Medicine, Taipei Medical University, 250 Wu-Hsing Street, Taipei 11031, Taiwan; 10 Institute of Biotechnology, National Taiwan University, 1 Roosevelt Road, Sec. 4, Taipei 10617, Taiwan; 11 Department of Medical Research, China Medical University Hospital, China Medical University, 91 Hsueh-Shih Road, Taichung 40402, Taiwan; 12 Department of Optometry, Asia University, 500 Lioufeng Rd., Wufeng, Taichung 41354, Taiwan

**Keywords:** kombucha, response surface methodology, glucuronic acid, antioxidation, SCOBY

## Abstract

Kombucha, a functional beverage rich in glucuronic acid, is fermented in the presence of acetic acid bacteria and yeast as the primary microorganisms. Glucuronic acid is recognized for its various physiological benefits, such as detoxification, antioxidation, and anti-inflammation. To optimize the glucuronic acid content in kombucha, various strain combinations by selecting fermented sources were accomplished. According to the experimental results, kombucha produced through co-fermentation with *Pichia anomala* and *Komagataeibacter hansenii*, with glucose-added black tea as the carbon source, exhibited the highest glucuronic acid production. A response surface methodology found that under optimized conditions of a 12.27% (*w*/*v*) carbon source concentration, a 10.07% (*w*/*v*) substrate concentration, and a 28.4 °C temperature, the highest glucuronic acid production reached 80.16 g/L, which represented a 2.39-fold increase compared to the original kombucha. Furthermore, the total polyphenol content increased by 3.87-fold, while DPPH and ABTS free radical–scavenging capacities increased by 1.86- and 2.22-fold, respectively. To sum up, these observations reveal the potential for commercial production of glucuronic acid–enriched kombucha and contribute to the development of functional food products related to kombucha in the future.

## 1. Introduction

Traditional kombucha, containing a symbiotic culture of acetic acid bacteria (AABs) and a yeast called SCOBY (symbiotic culture of bacteria and yeast), is fermented in sugared tea to produce a slightly acidic, fruity, aromatic beverage [1]. The major AABs involved in kombucha are specific genera such as *Acetobacter* spp., *Gluconobacter* spp., *Gluconacetobacter* spp., and *Komagataeibacter* spp. [2,3]. The major yeasts that are used present a wide array of strains suitable for the kombucha brewing process [4], including *Kloeckera*, *Pichia*, *Saccharomyces*, *Saccharomycoides*, *Shizosaccharomyces*, *Torulospora*, and *Zygosaccharomyces* [5,6,7,8]. The preferred traits of yeast species crucial for kombucha fermentation comprise osmotolerance, resilience to acidic conditions, and the ability to produce alcohol and various flavor compounds [4]. Interference among various yeast and AAB species can either enhance or disrupt their growth, and their metabolic characteristics may impact the characterizations and quality of the kombucha produced [9]. This suggests that the combination of different strains may have a certain potential impact on fermentation outcomes [10], and it is worth further selecting different combinations of yeast and AABs to optimize the biological functions and qualities of kombucha [11].

In addition to the microorganisms used, the quality of kombucha is also influenced by various fermentation conditions [12], such as the choice of fermentation substrate [13], the type and quantity of the carbon source [14], the temperature of the culture environment, and the duration of fermentation. During kombucha fermentation, yeast utilizes its own enzymes to hydrolyze sucrose into glucose and fructose. Subsequently, it produces ethanol through the process of glycolysis, while acetic acid bacteria make use of the glucose to produce gluconic acid and convert ethanol into acetic acid and other organic acids [15,16,17]. It is also worth noting that AABs may produce bacterial cellulose (BC) that floats on the liquid surface, which provides abundant dietary fiber and a special chewing texture, which is also a byproduct with unique value from kombucha.

Consuming kombucha offers a plethora of health benefits, aligning with its diverse biological functions and active ingredients, such as polyphenols, vitamins, and organic acids [18]. It was observed to lower blood pressure through angiotensin-converting enzyme inhibition, regulate blood sugar and cholesterol levels in vivo [19], and aid in weight management by controlling appetite through hypolipidemic effects in vivo and in vitro [20]. Polyphenols, known for their powerful antioxidant properties, significantly enhance the antioxidant and anti-obesity capacities of kombucha [21]. Polyphenols in tea-based kombucha primarily consist of flavonoids, flavanols, flavanol gallate, and flavanol glycosides [22]. Vitamins, essential for various biochemical and physiological processes in the body, cannot be synthesized internally. Therefore, maintaining healthy levels requires their supplementation through a balanced diet [23]. Kombucha was reported to contain water-soluble vitamins, including vitamin C and a spectrum of B vitamins (thiamine, riboflavin, niacin, pantothenic acid, vitamin B6, biotin, vitamin B9, and cobalamin) [24]. Kombucha fermentation produces vital organic acids like glucuronic, gluconic, citric, tartaric, folic, malonic, succinic, and pyruvic acids [25]. Notably, glucuronic acid, formed through microbial glucose oxidation, can mimic the detoxifying role of the human liver and acts as a precursor for vitamin C by its transformation into glucosamine in the body [14,26,27].

Nevertheless, to the present, most kombucha production processes utilized commercial SCOBY powder or kombucha liquid with ambiguous compositions as the starter culture for fermentation, which was revealed to lack standardization of microbial strains [28]. Standardizing microbial strains can contribute to the quality management of kombucha production, and when coupled with appropriate cultivation conditions, it can facilitate optimization of the health benefits of kombucha [29]. Therefore, it is essential to assess the use of co-cultured strains and optimize cultivation conditions. In this study, we established optimal conditions with selected SCOBY combinations for kombucha production. The metabolite, glucuronic acid, was selected because it is more representative due to its high antioxidation, as an indicator for assessing the degree of fermentation during kombucha production. The ultimate goal was to enhance the antioxidant ability of kombucha by utilizing an optimized fermentation process with an optimized combination of bacterial strains.

## 2. Materials and Methods

### 2.1. Materials and Microorganisms

The microbial strains, including *Komagataeibacter xylinus* ATCC 23767 and *Komagataeibacter hansenii* ATCC 23769, used in this study were obtained from the Bioresource Collection and Research Center (BCRC) (Hsinchu, Taiwan). *Saccharomyces cerevisiae*, *Pichia anomala*, and *Pichia kudriavzevii* were obtained from Prof. Cheng’s lab (Taipei, Taiwan). Dried leaves of *Camellia sinensis* L. (black tea) were sourced from HerbSunny (Nantou, Taiwan). Peptone and bacto agar were purchased from Bioshop (Ontario, Canada), while yeast extract was purchased from Biolife (Milan, Italy). Glucuronic acid, 2,2′-azino-bis (3-ethylbenzothiazoline-6-sulphonic acid) (ABTS), 2,2-diphenyl-1picryl-hydrazyl (DPPH), and Folin–Ciocalteu’s phenol were acquired from Sigma-Aldrich (St. Louis, MO, USA).

### 2.2. Preparation of Kombucha Tea

After boiling 1 L of distilled water, *C. sinensis* (1%, *w*/*v*) was added as a substrate, and glucose (10%, *w*/*v*) was used as a carbon source. The mixture was soaked for 15 min, and then filtered. Subsequently, it was sterilized at 121 °C for 15 min and cooled to room temperature for use. Then, 5% activated bacteria (yeast: acetic acid bacteria = 1:1) were inoculated into 60 mL of black tea, followed by culturing at 28 °C for 10 days.

### 2.3. Analysis of Glucuronic Acid

The high-performance liquid chromatographic (HPLC) (Jasco Inc., Tokyo, Japan) method employed in this study was adapted from Jayabalan et al. [30] with modifications. Samples were centrifuged at 10,000× *g* for 10 min at 4 °C. The supernatant was filtered using a 0.45-μm pore size filter. Afterwards, the HPLC analysis was conducted using a SynergiM4 um Hydro-RP80A column (4.6 × 250 mm) at 22 °C, with an injection volume of 10 μL and a flow rate of 0.5 min/mL. The mobile phase consisted of 20 mM KH_2_PO_4_ (Sigma-Aldrich, St. Louis, MO, USA) at pH 2.4. Glucuronic acid (Sigma-Aldrich, St. Louis, MO, USA) was quantitatively analyzed based on the standard curve of the standard solution.

### 2.4. Response Surface Methodology

The Box–Behnken design (BBD) of the response surface method (RSM) was established for 17 random runs, to determine optimized kombucha conditions that maximized glucuronic acid production. Factor standardization was X_1_ (glucose), X_2_ (black tea), and X_3_ (temperature) (Table 1), and the experimental design center was performed with five replicates. The equation of the quadratic model was represented by the coefficient of determination (*R*^2^), and its statistical significance was tested by the Fischer test (*p* < 0.05). An analysis of variance (ANOVA) was performed to check the compatibility of the proposed model with the experimental data.

### 2.5. Analysis of Metabolites

A modified version of the HPLC method proposed by Lončar, et al. [31] was used. Samples underwent centrifugation at 10,000× *g* for 10 min at 4 °C. The supernatant was filtered through a 0.45-μm pore size filter. Afterwards, HPLC was used for the analysis using a Repro-GelCatt column (9 μm, 300 mm × 8 mm) at 35 °C. The injection volume was 10 μL, and the flow rate was set to 0.5 mL/min. The mobile phase was comprised of 5 mM H_2_SO_4_. Sucrose, glucose, fructose, and ethanol were quantitatively analyzed based on the standard curve of the respective standards.

The total phenol content (TPC) in kombucha samples was determined using a colorimetric method described by Ahmed, et al. [32] with slight modification. Folin–Ciocalteu reagent (at 50 μL) was added to 50 μL of each sample solution, and the mixture was thoroughly mixed for 3 min at room temperature. Next, 50 μL of 2% Na_2_CO_3_ solution was slowly added, and the reaction mixture was placed in the dark for 2 h. The absorbance of the mixture was determined at 760 nm with an enzyme-linked immunosorbent assay (ELISA) reader. The TPC was then calculated using a gallic acid standard curve and expressed as milligrams of gallic acid equivalents per milliliter (mg GAE/mL).

### 2.6. Determination of pH Values

pH values of the kombucha samples were measured with a commercial pH meter (Denver Instruments, Bohemia, NY, USA). The pH meter was calibrated at pH 4.0, pH 7.0, and pH 10.0 before use.

### 2.7. Analysis of the Antioxidant Capacity

The DPPH free radical–scavenging ability of kombucha was evaluated following a method of Jayabalan et al. [33] with slight modification. In this procedure, 20 μL of each sample solution was mixed with 180 μL of a 20 mM DPPH solution in 80% ethanol, and the reaction mixture was kept in the dark for 20 min. The absorbance of DPPH was measured at 517 nm on an ELISA reader. The DPPH free radical–scavenging ability was calculated according to the following equation:

DPPH free radical–scavenging ability (%) = [1 − (*As* − *Ab*)/*Ac*] × 100 (1)

where *As* is sample +DPPH reagent, *AC* is ddH_2_O +DPPH reagent, *Ab* is sample +ddH_2_O, and different concentrations of ascorbic acid were additionally prepared as a control group.

The ABTS free radical–scavenging ability of kombucha was evaluated following a method outlined by Ilyasov, et al. [34] with slight modification. In this procedure, a 7 mM ABTS solution was mixed with an equivalent volume of a 2.45 mM K_2_S_2_O_8_ solution, and the mixture was kept in the dark for at least 12 h before use. Thereafter, the mixture was diluted to an absorbance of 0.70 ± 0.02 at 734 nm. Then, 20 μL of each sample solution was mixed with 180 μL of a dilute ABTS solution, and the reaction mixture was placed in the dark for 6 min. The absorbance of ABTS was measured at 734 nm on an ELISA reader. The ABTS free radical–scavenging ability was calculated according to the following equation:

ABTS free radical–scavenging ability (%) = [1 − (*As* − *Ab*)/*Ac*] × 100 (2)

where *As* is sample +ABTS reagent, *Ab* is sample +ddH_2_O, *Ac* is ddH_2_O +ABTS reagent, and different concentrations of ascorbic acid were additionally prepared as the control group.

### 2.8. Analysis of Microbiota Location from the Produced Bacterial Cellulose (BC)

The BC produced between the medium–air interface of kombucha was collected in a flask. Then, 10 µL of a mixture of concanavalin A and DAPI fluorescent dye was added, and was incubated for 20 min in the dark. Following that, a microscopic examination was conducted, and images were captured using a confocal laser scanning microscope (CLSM) (Leica TCS SP5, Leica, Microsystems, Wetzlar, Germany).

### 2.9. Statistical Analysis

All experiments were conducted at least in triplicate, and the data obtained are expressed as the mean ± standard deviation (SD). Statistical analysis was performed using the SPSS 23.0 software package (IBM, Armonk, NY, USA). The data underwent an analysis of variance (ANOVA), and significant differences between means were determined by Duncan’s multiple-range test at a significance level of *p* < 0.05. Optimization of fermentation conditions and the main composition analysis were carried out using Minitab vers. 19 software (Minitab, PA, USA).

## 3. Results and Discussion

### 3.1. Different SCOBY Combinations in Kombucha Production

**Figure 1:**
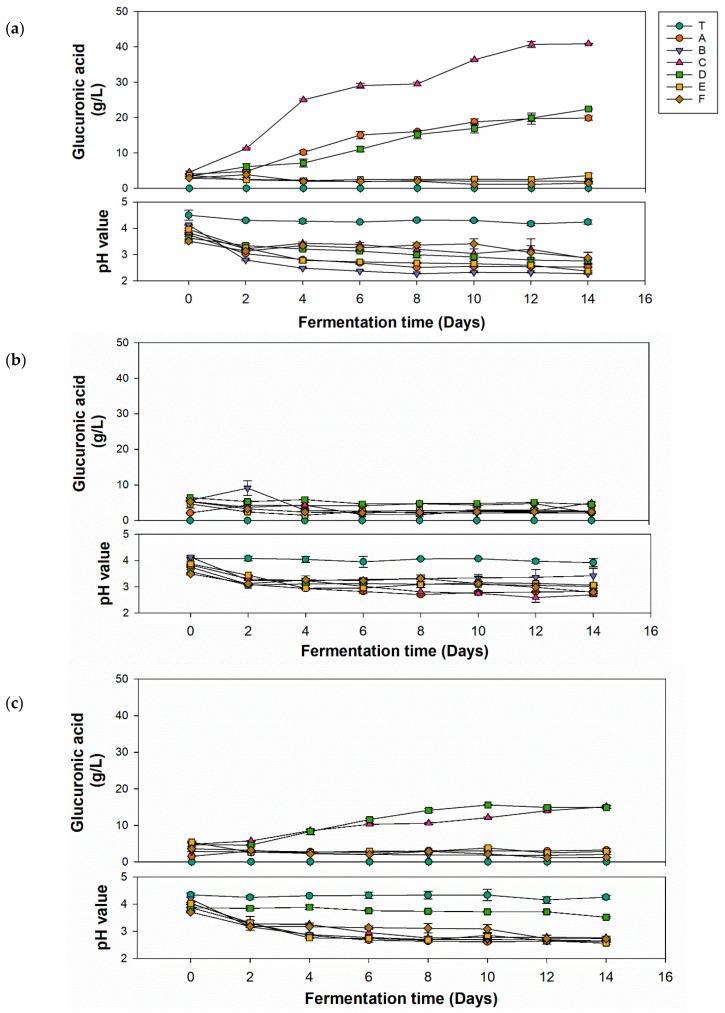
Glucuronic acid production and pH value change of kombucha with different strains using different carbon sources of (**a**) glucose, (**b**) fructose, and (**c**) sucrose. (T, black tea; A, *Pichia kudriavzevii* + *Komagataeibacter hansenii*; B, *P. kudriavzevii* + *K. xylinus*; C, *P. anomala* + *K. hansenii*; D, *P. anomala* + *K. xylinus*; E, *Saccharomyces cerevisiae* + *K. hansenii*; F, *S. cerevisiae* + *K. xylinus*).

Most kombucha-related studies utilize mixed microbial powders as the inoculum source, which not only makes it impossible to identify the specific microorganisms and their proportions, but also hinders comparisons between studies from different research group. Therefore, this study employed identified microorganisms as the inoculum source, using specific inoculation ratios for kombucha fermentation. This approach is beneficial in providing relevant information for the standardized production of kombucha. Figure 1 shows the production of glucuronic acid and variations of pH values in kombucha fermented with different carbon sources for 14 days. By comparing the three carbon sources, the group using glucose (Figure 1a) had the highest glucuronic acid yield (40.85 g/L) utilizing *P. anomala* with *K. hansenii*, which was double the quantity produced by the second and third highest combinations. The group that used fructose (Figure 1b) showed a nonsignificant increase in the glucuronic acid yield in all strain combinations. The group that used sucrose (Figure 1c) exhibited a slight increase in glucuronic acid yield to 15.5 m (*P. anomala* with *K. xylinus*). The pathway of glucuronic acid formation involves several steps. Sucrose is hydrolyzed into glucose by yeast, and glucose is then converted into glucuronic acid by AABs. Therefore, glucose and sucrose can be directly used in the biosynthesis pathway [35]. However, fructose needs to be converted to glucose via isomerase, which is lacking in yeast and AABs. This might be the reason why fructose as the carbon source showed lower yields of glucuronic acid. Based on these experimental results, subsequent optimization experiments utilized glucose as the carbon source for kombucha production.

The decline in pH during fermentation of kombucha was primarily attributed to the interaction between the AABs and yeast, leading to the generation of organic acids, including glucuronic acid. As depicted in Figure 1a–c, most of the pH values of different combinations significantly decreased after 4 days of fermentation and remained relatively stable thereafter until the end of fermentation. These results were due to the buffering effect caused by the synthesis of organic acids. Organic acids in the fermentation solution reacted with minerals via carbon dioxide to produce zwitterions, which then reacted with hydrogen ions in the organic acids, preventing the accumulation of hydrogen ions and maintaining the buffering capacity of the fermentation solution [36]. In Figure 1a, except for unfermented tea, pH values of other groups significantly dropped to 2.79–3.34 after the second day, then stabilized, with the final pH ranging 2.27–2.87 at the end of fermentation. In Figure 1b, the pH of the unfermented tea remained nearly unchanged over 14 days, while the pH of other groups had dropped to 3.06–3.44 by the second day and then stabilized, with the final pH ranging 2.69–3.42. In Figure 1c, the unfermented tea’s pH remained nearly unchanged over 14 days. Group D did not experience a sharp pH drop in the first 2 days like other groups, and showed a stable trend from an initial pH of 3.86 to a final pH of 3.51. The initial pH of other groups was 3.7–4.02, and the final pH ranged 2.56–2.76. For kombucha to be considered safe according to US Food and Drug Administration (FDA) regulations for acidic foods, its pH must be between 2.5 and 4.2. If the pH does not reach ≤4.2 within 7 days of fermentation, the culture is likely contaminated and should be disposed of properly. If the pH falls below 2.5, fresh tea should be added before consumption to avoid an overly sour taste and potential oral discomfort [37]. In brief, the kombucha produced in this experiment using glucose, fructose, and sucrose complied with FDA regulations. Only a few samples had a final fermentation pH below 2.5, which would need adjusting before being suitable for consumption as a product.

**Figure 2:**
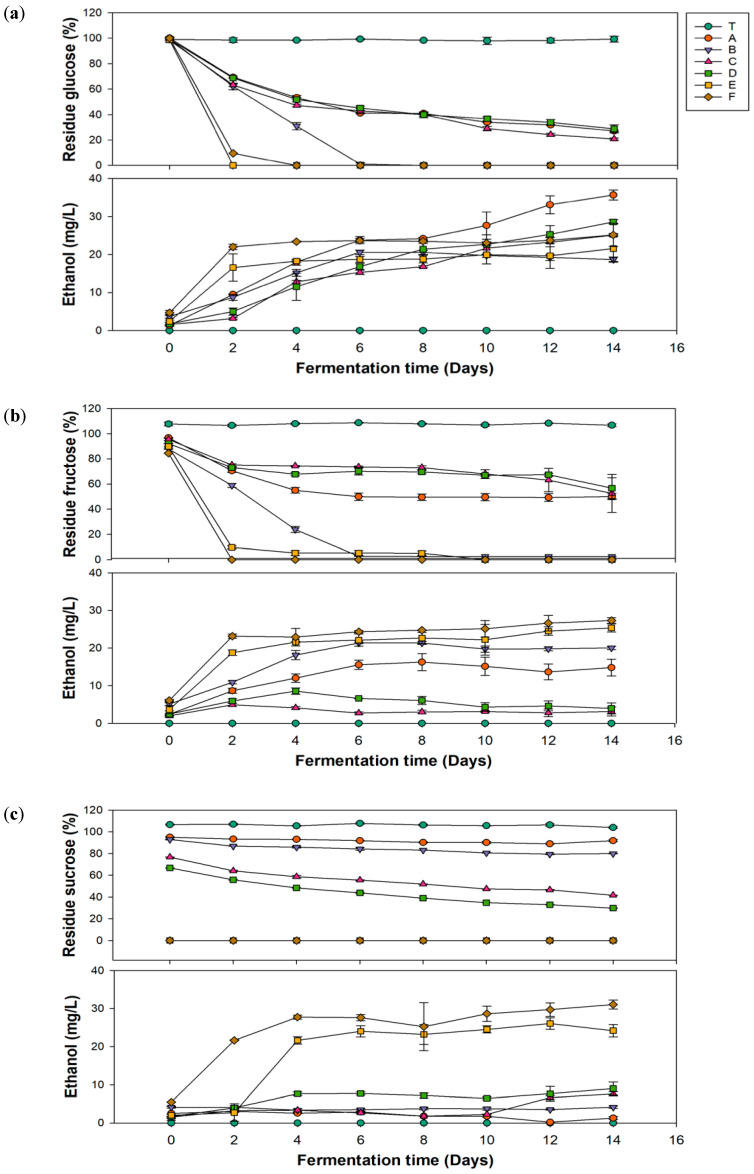
Changes in the residual sugar and ethanol contents during fermentation of kombucha using different carbon sources of (**a**) glucose, (**b**) fructose, and (**c**) sucrose. (T, black tea; A, *Pichia kudriavzevii* + *Komagataeibacter hansenii*; B, *P. kudriavzevii* + *K. xylinus*; C, *P. anomala* + *K. hansenii*; D, *P. anomala* + *K. xylinus*; E, *Saccharomyces cerevisiae* + *K. hansenii*; F, *S. cerevisiae* + *K. xylinus*).

In Figure 2a, group C, which had the highest glucuronic acid production, showed a decrease in the glucose concentration to 47.19 mg/L on the 4th day of fermentation, ultimately reaching 20.83 mg/L on the 14th day. Groups E and F exhibited the most dramatic decreases in glucose concentrations during the first 2 days of fermentation, with no residual sugar remaining after the fourth day. The residual sugar content affects the final sweetness of kombucha. Generally, kombucha emphasizes health benefits; excessive residual sugar may lead to continued fermentation after shelf placement, causing an increase in the alcohol content. Additionally, excessive intake of simple sugars can result in blood sugar fluctuations, stimulate insulin secretion, and potentially lead to insulin resistance, which indirectly contributes to fat accumulation and the occurrence of metabolic disorders. Therefore, controlling residual sugar is an important parameter to monitor. According to a 2023 study by Rukman and Haerussana [38], kombucha made with refined sugar, palm sugar, and honey had residual sugar levels of 51.95, 67.95, and 88.9 mg/L, respectively. Results of residual sugar contents in this study were relatively low, which aligns better with modern health demands.

Ethanol is a typical by-product of kombucha fermentation [39]. However, if the alcohol by volume (ABV) exceeds 0.5% during the fermentation process, it contradicts the perception of kombucha as a health beverage. Additionally, kombucha is often sold as a nonalcoholic drink, and an excessive alcohol content can adversely affect specific groups, such as pregnant women and children [39]. Therefore, monitoring the ethanol content is crucial. In Figure 2b, groups E and F showed sharp increases in ethanol during the first 2 days of fermentation, reaching 16.56 and 22 mg/L, respectively, and then remained stable. The ethanol content in groups A–D gradually increased with the fermentation time, peaking on the 14th day, with group A reaching the highest level of 35.69 mg/L.

Based on the experimental data, the alcohol content of kombucha produced in this study was far below 0.5%. In addition, as mentioned by regulations in Taiwan and the United States, beverages with alcohol concentrations exceeding 0.5% are classified as alcoholic beverages and are subject to related taxation. Although alcoholic versions of kombucha are available in European markets, it is important to consider alcohol content adjustments due to their impact on production costs. In line with Jang, McIntyre, Chan, Brown, Finley, and Chen [39] in a 2021 survey, some commercially available kombucha in the United Kingdom has an average alcohol content of approximately 0.77%, with about 31.5% of them having an alcohol content exceeding 1%. In comparison, the kombucha produced in this study not only better meets health demands but also effectively reduces potential production costs.

### 3.2. Optimal Single-Factor Fermentation Conditions for Gluconic Acid Production

**Figure 3:**
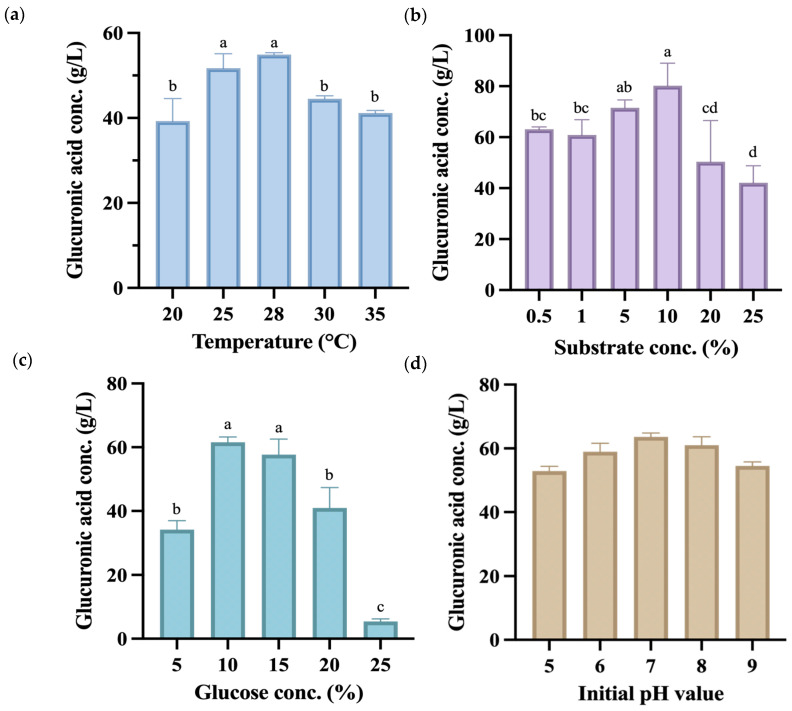
Glucuronic acid (g/L) content during the 14-day fermentation process of kombucha produced using glucose as a carbon source under different fermentation conditions, including (**a**) temperature, (**b**) substrate concentration, (**c**) glucose concentration, and (**d**) initial pH value. Different letters on the error bars indicate a significant difference (*p* < 0.05).

Subsequent single-factor fermentation experiments used the co-fermentation group of *P. anomala* and *K. hansenii* to produce kombucha fermented for 14 days, with the endpoint samples analyzed. As shown in Figure 3a, the highest glucuronic acid yield was achieved at 28 °C, reaching 54.92 g/L, which was markedly higher than yields at other fermentation temperatures. All temperature had significant effects (*p* < 0.05), along with the interaction with glucuronic acid production. However, a related study reported that the temperature at which kombucha produces the highest amount of glucuronic acid is 30 °C, with a concentration of 48.0 g/L [40].

The experiment used black tea made from *C. sinensis* that contains various compounds such as polyphenols, flavonols, catechins, adenine, and caffeine, which contribute to its strong antioxidant properties [41]. During the manufacture of black tea, *C. sinensis* leaves undergo processing that activates polyphenol oxidases, leading to the oxidation of catechins. This process forms dimers and polymers known as theaflavins and thearubigins, the primary polyphenolic compounds in black tea [42]. As depicted in Figure 3b, when utilizing a substrate concentration of 10% (*w*/*v*), the kombucha demonstrated a glucuronic acid production of 80.16 g/L, which was significantly higher than other substrate concentrations produced. Hence, this concentration was selected for subsequent use as a factor and central point in the response surface methodology.

Glucuronic acid is formed through the glucuronidation of the sixth carbon of glucose. Therefore, during the cultivation process, increasing the concentration of glucose in the culture medium can effectively enhance the yield of glucuronic acid [43]. Nevertheless, excessive addition of glucose might lead to an elevated osmotic pressure, which could unfavorably impact microbial growth. Figure 3c shows that there was no statistically significant difference between the 10% (*w*/*v*) and 15% (*w*/*v*) groups, as significantly higher glucuronic acid yields were seen at both concentrations compared to other groups. Based on this experimental finding, a carbon source concentration of 12.5% (*w*/*v*) was selected for subsequent response surface methodology as the factor and central point.

The initial pH value of kombucha before fermentation is typically around 5.5 [44]. A previous study indicated that a higher initial pH value increases acid production while decreasing cellulose production [45]. Therefore, this experiment adjusted the initial pH to values between 5 and 9 to observe whether it affected the production of glucuronic acid. Figure 3d shows that at an initial pH value of 7, the highest amount of glucuronic acid was produced after 14 days of fermentation. Despite that, there were no significant differences between the groups, indicating that the initial pH value does not significantly impact glucuronic acid production. Consequently, the initial pH value was not controlled in subsequent experiments. Results of the single-factor experiment were used to select a suitable central point condition in the following RSM experiment.

### 3.3. Optimization of Glucuronic Acid Production Using RSM

The optimization of glucuronic acid production through single-factor fermentation conditions included varying the glucose concentration (X_1_), black tea concentration (X_2_), and fermentation temperature (X_3_) (Table 1). These three factors were used as variables in the BBD model (Table 2). The mathematical model for the optimization of glucuronic acid production using RSM was as follows:

Y = −430.8 + 6.95 X_1_ + 8.32 X_2_ + 30.11 X_3_ − 0.4351 X_1_^2^ − 0.5201 X_2_^2^ − 0.5879 X_3_^2^ − 0.1099 X_1_X_2_ + 0.1688 X_1_X_3_ + 0.1225 X_2_X_3_. (3)

Results of the analysis of the variance table for the fit regression model on optimized kombucha are shown in Table 3. The lack-of-fit test for the model yielded a value of 0.175, indicating statistical reliability. The linear terms of the three factors (X_1_, X_2_, and X_3_) did not show significant effects. However, the squared terms of all three factors had significant effects on the model (*p* < 0.05). Among the interactions between the three factors, only the interaction between the carbon source concentration and temperature was significant (*p* < 0.05). Therefore, the prediction equation was regulated by applying a regression analysis of actual variables in Equation (4):

Y = −430.8 − 0.4351 X_1_^2^ − 0.5201 X_2_^2^ − 0.5879 X_3_^2^ + 0.1688 X_1_X_3_. (4)

**Figure 4:**
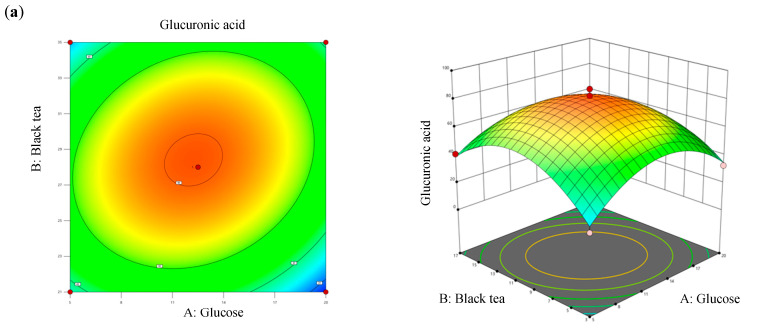

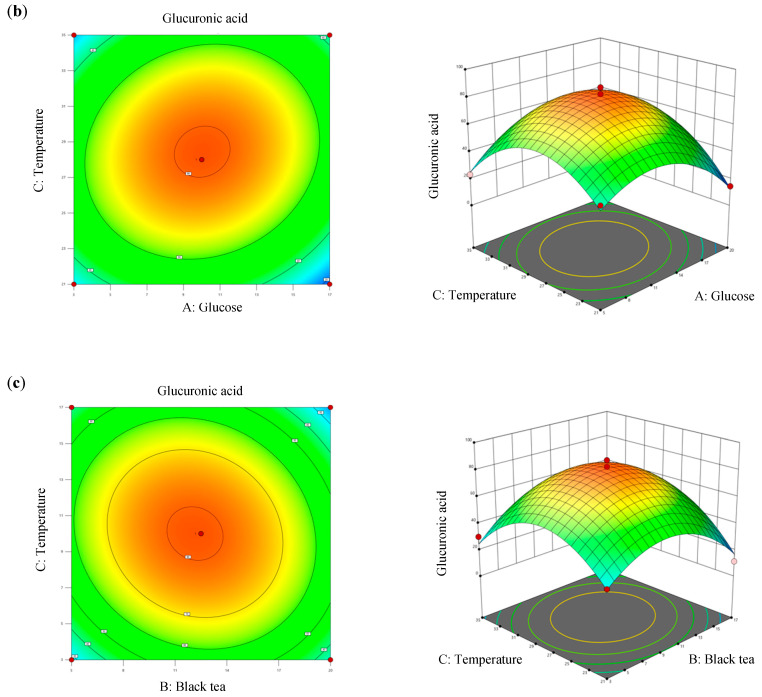
Three-dimensional surface of glucuronic acid production showing interactions among the three tested variables: (**a**) glucose and black tea, (**b**) glucose and temperature, and (**c**) black tea and temperature.

Values of *R*^2^ and adjusted *R*^2^ of the model differed by 2.4%, which was less than 20%, denoting that all three factors significantly contributed to the optimization equation. Additionally, an adjusted *R*^2^ value of >0.8 met the criteria for optimal applicability in a second-order model. Figure 4 illustrates contour plots and response surface plots for glucuronic acid production. Calculations based on the model identified the optimal fermentation conditions for maximum glucuronic acid production as follows: a glucose concentration of 12.27% (*w*/*v*), a black tea concentration of 10.07% (*w*/*v*), and a temperature of 28.4 °C. Under these conditions, the theoretical maximum glucuronic acid production was 81.24 g/L, which was 2.39-times higher than that of the original kombucha fermentation (Table 4).

A previous study regulated the synthetic microbial community to obtain the highest acid production and found that the combination optimization strategy for microbial consortia can significantly increase the gluconic acid yield in kombucha [46].

### 3.4. Antioxidant Capacity and TPC of Optimized Kombucha

**Figure 5:**
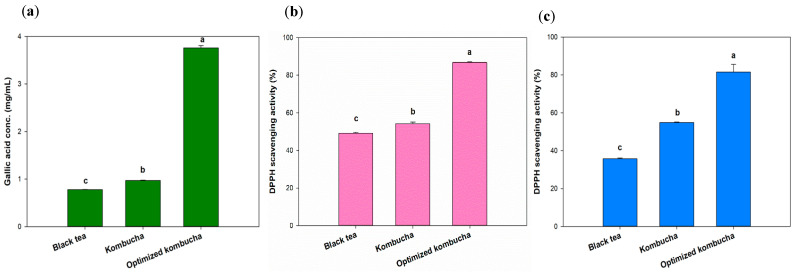
Total phenolic content (**a**) and antioxidant effects against DPPH (**b**) and ABTS (**c**) radicals. Different letters on the error bars indicate a significant difference (*p* < 0.05).

Tea is known to contain polyphenols with strong antioxidant properties. Interactions between phytochemicals and changes in the structure of tea phenolics, such as catechins (especially (−) epigallocatechin gallate), caused by microbial enzymes, as well as the production of polyphenols, flavonoids, organic acids, and vitamin C [47], alter the antioxidant capacity after fermentation [48]. Yang et al. analyzed nine top-selling kombucha products. Among them, Better Booch Ginger Boost had the highest total polyphenol content, measured at 0.38 mg/mL of gallic acid [49]. Numerous studies confirmed that polyphenolic compounds possess antioxidative properties, thereby reducing free radical activity and oxidative stress [48]. Compared to those products, the kombucha optimized in this experiment exhibited a higher total polyphenol content, thus providing a greater antioxidant capacity. The black tea and unoptimized kombucha showed the similar GAE concentration of 0.78 and 0.97 mg/mL, which are as same as previous study [42]. The kombucha fermented under optimized conditions had a GAE concentration of 3.76 mg/mL, an almost 4-fold increase compared to the original fermentation group and unfermented group (Figure 5a).

The DPPH and ABTS antioxidant capacities of the optimized kombucha group were higher than those of the original fermentation group and the unfermented group (Figure 5b). In particular, the ABTS antioxidant capacity in the optimized group reached 78.75%, which was about 2.22-times higher than the original fermentation group (35.45%) and 3.39-times higher than the unfermented group (23.22%). Regarding the DPPH antioxidant capacity, the overall free radical–scavenging rate was lower compared to ABTS. However, the optimized group (12.44%) still demonstrated a 1.86-fold increase compared to the original fermentation group (6.69%) and a 2.14-fold increase compared to the unfermented group (5.80%). ABTS and DPPH assays are respectively used to measure water-soluble and fat-soluble antioxidants. These results suggested that the water-soluble bioactive compounds in kombucha may be more easily released or are present in higher amounts compared to fat-soluble bioactive compounds.

Previous studies [42] have shown that the antioxidant activity of kombucha is positively correlated with its polyphenol types and content. The result also demonstrated a similar trend that the polyphenol content in the optimized kombucha significantly increased, leading to a notable enhancement in antioxidant capacity of DPPH and ABTS. However, the trend may change in certain types of kombucha fermentation such as fruit-based kombucha. Some specific enzymes present in the fruit may degrade polyphenols, resulting in a decrease in polyphenol concentration but an increase in antioxidant activity [50].

### 3.5. Analysis of Microbiota Location from the Produced BC

**Figure 6:**
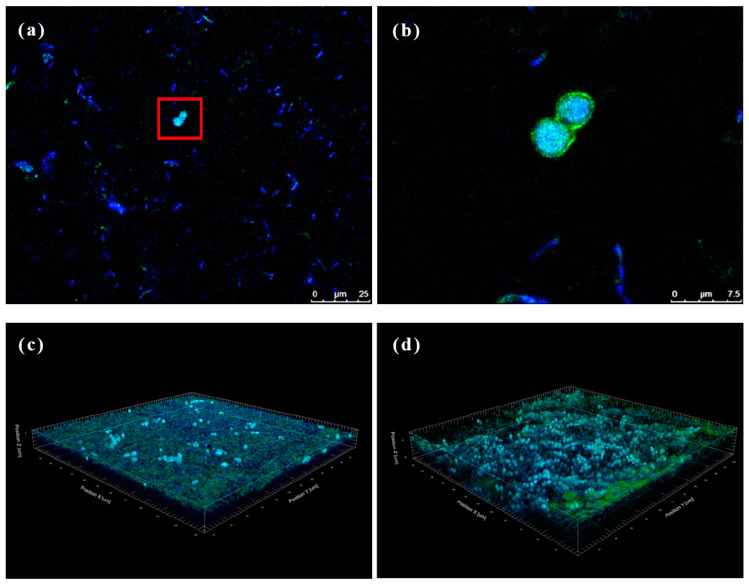
Immunofluorescence staining of bacterial cellulose (BC). (**a**) Upper layer region from optimized kombucha, (**b**) an enlarged view of the area outlined, and (**c**) upper and (**d**) lower regions of the 3D modeling of BC.

Kombucha is primarily produced through the co-fermentation of AABs and yeast. However, BC formed on the surface of kombucha is solely produced by AABs, with no clear evidence of yeast’s influence. This experiment aimed to observe the BC microbiota using CLSM, providing insights into the microbial composition and characteristics of optimized kombucha. Figure 6 shows that both dyes (concanavalin A and DAPI) exhibited their respective fluorescence colors. Concanavalin A stained the yeast cell membranes green (see the red frame in Figure 6a), while DAPI dyed the DNA of all cells blue. Sectioning techniques revealed that yeast cells were detected at depths of approximately 3–10 μm in both the upper and lower layers of the BC, and *Acetobacter* was found at depths ranging 0.5–20 μm. The distribution of *Acetobacter* in the upper and lower layers of the BC showed no significant difference, but yeast cells appeared denser in the lower layer, indicating a higher concentration of yeast cells in the lower layer. BC is produced by AABs from top to bottom during kombucha fermentation [51]. The yeast might be blocked from the underside layer due to the nanoscale network structure of BC, which therefore results in a higher growth density of yeast in the lower layer. Uneven bacterial symbiosis may affect the material properties of BC and should be further evaluated in future work.

## 4. Conclusions

Most studies on kombucha use mixed cultures as the inoculum, which results in unclear inoculation concentrations and microbial species. This may make it challenging to compare the yield of specific products under identical conditions in related research data.This study used identified microbial species and specific inoculation concentrations for kombucha fermentation, thus providing valuable information for a standardized process.

The effects of various variables in the optimized kombucha fermentation conditions on glucuronic acid production, antioxidant activity, and TPC were investigated. The combination of *P. anomala* and *K. hansenii* as co-fermentation strains achieved the highest glucuronic acid production among six different co-fermentation experimental groups. After optimizing the fermentation conditions through RSM, glucuronic acid production reached 80.16 g/L, significantly higher than previously reported values for kombucha glucuronic acid production. This marked a substantial 2.39-fold improvement compared to the original kombucha fermentation process. Moreover, the RSM-optimized kombucha exhibited an impressive 3.87-fold increase in TPC compared to the original fermentation group. Additionally, it showed significant enhancements in both ABTS and DPPH free radical–scavenging capacities, with respective increases of 2.22-fold and 1.86-fold. In summary, the application of RSM significantly boosted the glucuronic acid production, antioxidant capacity, and TPC of kombucha. However, further research is needed to explore the effects of kombucha optimized for glucuronic acid production on specific health benefits, as well as the prevention and treatment of diseases, particularly in cell-based studies and clinical settings, with a focus on metabolic syndrome–related conditions.

## Figures and Tables

**Table 1:** Coded levels and experimental values of the independent variables.

Factors	Symbol	Coded Variable Level		
		−1	0	1
Glucose (% (*w*/*v*))	X_1_	5	12.5	20
Black tea (% (*w*/*v*))	X_2_	3	10	17
Temperature (°C)	X_3_	21	28	35

**Table 2:** Comparisons of the actual value and theoretical value models of glucuronic acid production of optimized kombucha.

Run Order	X_1_	X_2_	X_3_	X_1_ Glucose (%, *w*/*v*)	X_2_ Black Tea (%, *w*/*v*)	X_3_ Temp. (°C)	Actual Value (g/L)	Theoretical Value (g/L)
1	1	0	1	20	10	35	34.61	38.08
2	−1	0	1	5	10	35	23.37	24.85
3	0	1	1	12.5	17	35	35.66	36.28
4	1	0	−1	20	10	21	14.52	13.04
5	0	0	0	12.5	10	28	77.26	81.09
6	−1	1	0	5	17	28	41.06	38.97
7	0	0	0	12.5	10	28	87.26	81.09
8	1	−1	0	20	3	28	32.73	34.83
9	0	1	−1	12.5	17	21	11.31	16.96
10	−1	−1	0	5	3	28	23.72	27.79
11	0	0	0	12.5	10	28	82.5	81.09
12	0	−1	−1	12.5	3	21	29.95	29.32
13	1	1	0	20	17	28	27.01	22.93
14	−1	0	−1	5	10	21	38.74	35.26
15	0	−1	1	12.5	3	35	30.28	24.63
16	0	0	0	12.5	10	28	80.5	81.09
17	0	0	0	12.5	10	28	77.92	81.09

**Table 3:** Analysis of the variance table for the fit regression model of optimized kombucha.

Factors	df	Sum of Squares	Mean Square	*F* Value	*p* Value
*Linear*					
X_1_	1	40.6	40.6	1.42	0.272
X_2_	1	0.3	0.34	0.01	0.917
X_3_	1	108.1	108.08	3.78	0.093
*Square*					
X_1_X_1_	1	2521.7	2521.75	88.25	0.000
X_2_X_2_	1	2734.7	2734.74	95.7	0.000
X_3_X_3_	1	3493.6	3493.6	122.26	0.000
*Interaction*					
X_1_X_2_	1	133	133.04	4.66	0.068
X_1_X_3_	1	314.2	314.23	11	0.013
X_2_X_3_	1	144.2	144.19	5.05	0.060
Lack-of-Fit	3	134.9	44.98	2.76	0.175
	coefficient δ				
*R* ^2^	0.9813				
Adjusted *R*^2^	0.9573				

**Table 4:** Maximum glucuronic acid production of original and optimized kombucha.

	Glucose (%, *w*/*v*)	Black Tea (%, *w*/*v*)	Temp. (°C)	Glucuronic Acid (g/L)	
				Actual Value	Theoretical Value
Kombucha	10	1	28	33.49	----
Optimized kombucha	12.27	10.07	28.4	80.16	81.24

## Data Availability

Data are contained within the article.
